# Restrained expression of canine glucocorticoid receptor splice variants α and P prognosticates fatal disease outcome in SIRS

**DOI:** 10.1038/s41598-021-03451-0

**Published:** 2021-12-30

**Authors:** Brigitta Margit Kállai, Judit Csöndes, Gergely Kiss, Lilla Bodrogi, Zsolt Rónai, Tamás Mészáros

**Affiliations:** 1 Department of Molecular Biology, Faculty of Medicine, Semmelweis University, Budapest, 1085 Hungary; 2 Department of Clinical Pathology and Oncology, University of Veterinary Medicine, Budapest, 1078 Hungary; 3 PraxisLab Ltd, Budapest, 1038 Hungary; 4 Department and Clinic of Internal Medicine, University of Veterinary Medicine, Budapest, 1078 Hungary; 5 Department of Animal Biotechnology, Institute of Genetics and Biotechnology, Hungarian University of Agriculture and Life Sciences, Gödöllő, 2100 Hungary

**Keywords:** Gene expression, RNA splicing, Sequencing, Molecular medicine, Transcription, Transcriptomics, Prognostic markers

## Abstract

Glucocorticoids play a central role in the inflammatory response and alleviate the symptoms in critically ill patients. The glucocorticoid action relies on the glucocorticoid receptor (GR) which translocates into the nucleus upon ligand-binding and regulates transcription of a battery of genes. Although the GR is encoded by a single gene, dozens of its splice variants have been described in diverse species. The GRα isoform encodes the full, functionally active protein that is composed of a transactivation, a DNA-binding, and a C-terminal ligand-binding domain. The second most highly expressed receptor variant, the GR-P, is formed by an intron retention that introduces an early stop codon and results in a probably dysfunctional protein with truncated ligand-binding domain. We described the canine ortholog of GR-P and showed that this splice variant is highly abundant in the peripheral blood of dogs. The level of cGRα and cGR-P transcripts are elevated in patients of SIRS and the survival rate is increased with elevated cGRα and cGR-P expression. The ratio of cGRα and cGR-P mRNA did not differ between the survivor and non-survivor patients; thus, the total GR expression is more pertinent than the relative expression of GR isoforms in assessment of the disease outcome.

## Introduction

Adequate glucocorticoid response is essential for alleviation of inflammation and restoration of vascular tone in critically ill patients^1–3^. Diverse biological actions of glucocorticoids are mediated via multiple glucocorticoid receptor (GR) subtypes. The human GR (hGR) protein-encoding *NR3C1* gene consists of nine exons, the first one is a translation regulator, while the remaining eight are translated into diverse protein isoforms. Although dozens of hGR splice variants have been described, no unambiguous physiological function has been assigned to most of them yet^4,5^.

The mRNA of the longest isoform of hGR, the hGRα codes for a functionally active protein of 777 amino acids that is composed of three major domains, i.e. a transactivation, a DNA-binding, and a C-terminal ligand-binding domain. The most extensively studied isoform is hGRβ that is the result of an alternative splicing of exon 9. In comparison to the hGRα, the β isoform consists of only 742 amino acids and possesses a unique 15 amino acid long C-terminal^6,7^. Although hGRβ is widely expressed in many cell types, its mRNA expression level is two–three orders of magnitude lower than that of hGRα^8–10^. The functional consequence of truncation and amino acid alteration of the C-terminal of the hGRβ receptor is a dysfunctional ligand-binding domain that is reported to exert a dominant-negative effect on the ligand-activated hGRα^11–13^. hGR-P (also known as hGR-δ) isoform is formed by the retention of the penultimate intron G that introduces a premature stop codon and its translation results in a protein consisting of 676 amino acids, which retains its DNA-binding domain but lacks an even larger segment of the ligand-binding domain than the β isoform^14–18^. Although the expression level of hGR-P mRNA is much higher than that of hGRβ, less research aimed to reveal the function of hGR-P. One of the few hGR-P studying publications showed that in contrast to hGRβ, this isoform enhanced the transcription promoting activity of hGRα^18^. This result is even more puzzling considering a more recently described splice variant which is formed by the retention of the last intron H (hGR-S1) and encodes a 745 amino acid long truncated protein that also demonstrated decreased transactivation potential^19^.

Besides human, GR isoforms produced by the retention of the last intron have been described in several animal species, e.g. zebrafish^20^, mouse^21^, rat^22^ and pig^23^. These variants have been referred to as β isoforms due to their structural and functional similarities to their human counterpart. Interestingly, the human orthologue of GR-P formed through the retention of the penultimate intron was only found in porcine tissues^23^ and more recently in mouse blastocysts^24^.

Alike its human homolog, the canine *NR3C1* gene contains nine exons. The canine *GR* gene is located on chromosome 2 and exons 2, 3–4 and 5–9 encode the N‐terminal transactivation domain, the DNA‐binding domain and the C-terminal ligand‐binding domain, respectively^25^. Costa et al. examined the liver transcriptome of ten healthy dogs by RNA-Seq and identified only two transcripts differing in their exon 1 coded 5’-untranslated region^25^. Another study analysed the whole blood cDNA of a dog with suspected iatrogenic Cushing’s syndrome by PCR amplification and downstream sequencing. The results suggested the presence of a novel GR splice isoform—designated cGRΔLBD—in which the penultimate intron 7 is only partially removed. The expression of this C-terminally truncated GR isoform drastically reduced glucocorticoid responsiveness of a glucocorticoid response element (GRE) driven vector in transiently transfected cells^26^.

Septic and non-septic systemic inflammatory response syndrome (SIRS) is a common cause of hospital admission in dogs and similarly to the human data, the mortality of the illness is as high as 21–68%^27–31^. Exaggerated pro-inflammatory cytokine production could lead to SIRS in critically ill patients with wide variety of clinical conditions regardless the primary disorder (septic or non-septic inflammation, trauma, neoplasia, etc.)^28,32^. SIRS can progress to multiple organ dysfunction syndrome^33^, central or peripheral circulatory disorders or coagulation abnormalities which finally leads to poor disease outcome, even with high-standard intensive care^31,34^. We aimed to study the putative involvement of GR hnRNA alternative splicing in canine SIRS by mRNA sequencing of a peripheral blood sample from a critically ill dog with septic peritonitis. Furthermore, we designed a real-time PCR approach to investigate the expression level of the GR mRNA in a cohort of healthy and critically ill dogs of various inflammatory disorders which represented an ordinary population of a small animal intensive care unit.

## Results

### Description of a novel canine GR isoform in peripheral blood of a patient with SIRS

**Figure 1:**
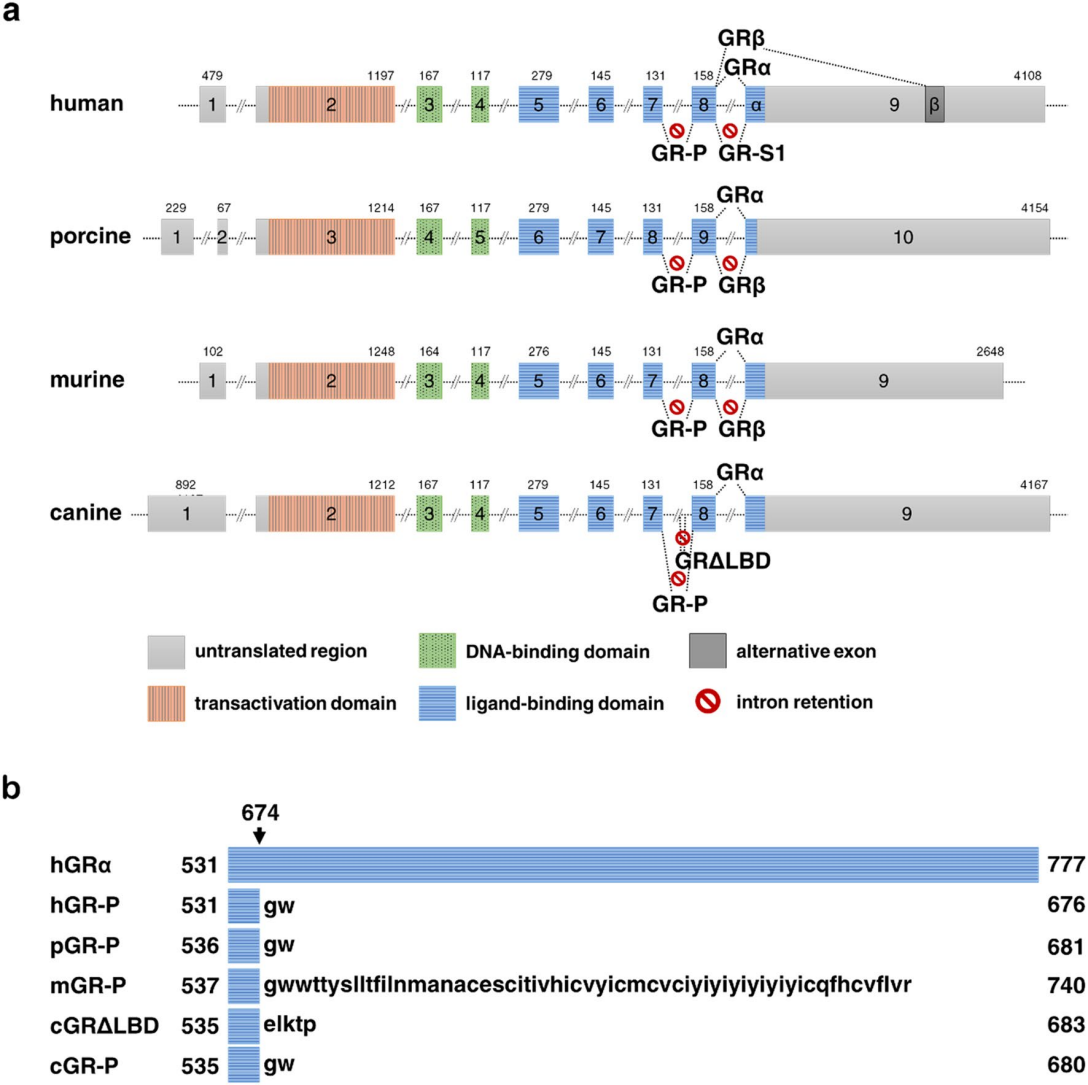
Schematic of the main 3’ end splice variants in human (h), porcine (p), murine (m) and canine (c) *NR3C1* gene and protein alignment of GR-P isoform of various species. **(a)** The main 3’ end splice variants of the human, porcine, murine and canine glucocorticoid receptor transcripts. Untranslated regions are marked by light grey boxes and the amino acid coding exons are coloured according to the encoded GR protein domains (i.e., orange: transactivation domain, green: DNA-binding domain, blue: ligand-binding domain, dark grey: alternative exon encoded amino acids). **(b)** Protein sequence alignment of the ligand-binding domain of GR-P isoform of different species.

Considering that altered expression of hGR mRNA isoforms was shown in several inflammatory diseases, we purified total RNA from peripheral blood of a critically ill dog with septic peritonitis (Patient 25 in Supplementary Table S1) to study whether SIRS triggers alternative splicing of the canine ortholog of *NR3C1* gene. The collected mRNA was analysed by next-generation sequencing and the obtained data showed that the expression level of cGRα mRNA is approximately two orders of magnitude lower than those of the most commonly used housekeeping genes such as β-actin and glyceraldehyde-3-phosphate dehydrogenase (data not shown). More interestingly, the alignment of the retrieved sequences to the CanFam3.1 reference genome definitely identified retention of intron 7 of the canine *NR3C1* in the cGR mRNA (Fig. 1a). Analysis of read distribution between introns and coding exons showed that the ratio of *NR3C1* intron 7 to all *NR3C1* exons was an order of magnitude higher than the global intron to exon ratio suggesting that the alternative mRNA transcript is a bona fide splice isoform (Supplementary Table S3). According to the mRNA-Seq data, this novel variant represented roughly 20% of the classical cGRα transcript pool (Supplementary Table S3). Next, we compared the nucleotide sequence of the mRNA-Seq obtained transcript to the corresponding canine GR genomic sequence and found 100% identity between all exons (data not shown) and 98% identity in the retained 8234 bp intronic sequence between exons 7 and 8 (Supplementary Fig. S1).

In silico translation of the intron 7 retaining splice variant identified an early stop codon at the beginning of the intron 7 leading to a 680 amino acid long protein. The truncated protein is 101 residues shorter than the wild-type glucocorticoid receptor and this shortening results in a partial loss of the ligand-binding domain (Fig. 1b). Considering the structural homology with the previously identified human and porcine GR-P isoforms (Fig. 1) we further refer to the identified truncated receptor as the canine cGR-P isoform. Alignment of cGR-P protein sequence to the formerly reported GR-P sequences of other species showed that it was 92, 90, and 80% identical to that of human, pig and mouse, respectively (Supplementary Fig. S2).

### Real-time PCR confirms the ubiquitous presence of GR-P ortholog in peripheral blood of dogs

**Figure 2:**
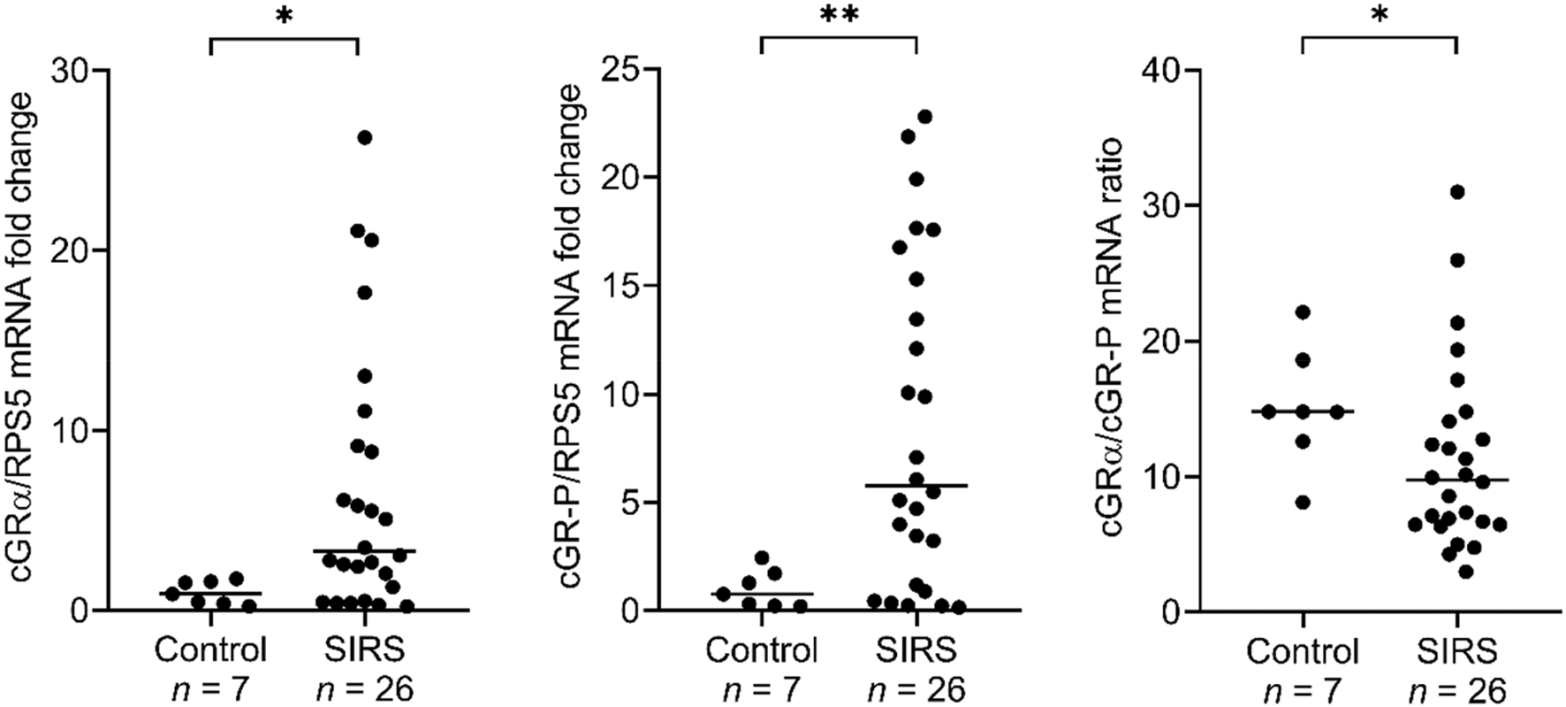
Real-time PCR analysis of cGRα and cGR-P mRNA expression and cGRα/cGR-P mRNA ratios in the control group and dogs suffering from SIRS. The number of dogs studied in each group is indicated under the graphs. cGRα and cGR-P mRNA expressions are normalized to reference gene *RPS5* expression and presented as fold changes compared to the mean of the control group. The lines represent the median values. Comparisons between two groups were performed by nonparametric Mann–Whitney *U* test. Statistically significant differences are depicted by *p < 0.05 and **p < 0.01.

**Figure 3:**
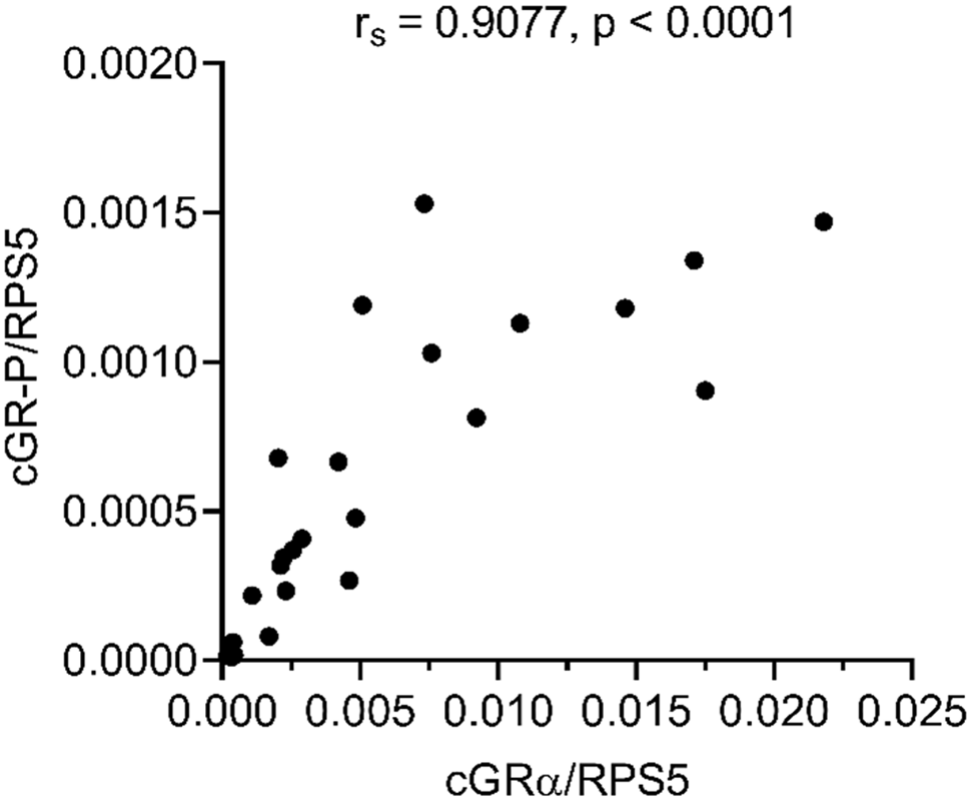
Correlation between cGRα and cGR-P mRNA expression in peripheral blood of SIRS patients at hospital admission. cGRα and cGR-P mRNA expressions are normalized to reference gene *RPS5* expression. Correlation analysis was performed by Spearman’s rank test and *p* < 0.05 was considered statistically significant.

The novel GR-P isoform was identified by using a sample obtained from a single critically ill dog and differed from the previously published canine GRΔLBD isoform^26^. Therefore, we aimed to confirm the mRNA-Seq results by investigating the occurrence of cGR-P using a TaqMan real-time PCR assay in a cohort of healthy dogs and patients suffering from SIRS. To this end, we designed a primer that could anneal to the junction of exon 6 and 7 of both isoforms. This primer was employed in combination with primers complementary to exon 8 and intron 7 to amplify the cGRα and cGR-P isoforms, respectively (Supplementary Fig. S3). The isoform-specific fluorescent probes could either bind to exon 7–8 junction site of cGRα or to the boundary of exon 7 and intron 7 in the case of cGR-P. The obtained real-time data confirmed the result of mRNA-Seq and clearly showed that both cGR isoforms could be found in peripheral blood samples of all dogs, i.e. including the healthy subjects (Fig. 2). The median mRNA expression of the classic cGRα variant and cGR-P splice variant was increased 3.5-fold (*p* = 0.0120) and 7.5-fold (*p* = 0.0067), respectively, in dogs with SIRS compared to healthy controls (Fig. 2), and a strong positive correlation (*r*_*s*_ = 0.9077, *p* < 0.0001) was observed between cGRα and cGR-P mRNA expression in the SIRS cohort (Fig. 3). Additionally, the ratio of median cGRα/cGR-P mRNA expression was significantly lower (*p* = 0.0497) in the SIRS group (9.77) than in the control group (14.80). Of note, the cGRα/cGR-P ratio varied between 3 and 31 amongst dogs in the analysed cohorts (Fig. 2).

### Correlation analysis of cGRα and cGR-P mRNA expression with various parameters of SIRS patients

Having confirmed the ubiquitous presence of cGRα and cGR-P and their elevated expression in SIRS patients, we set out to study putative correlations of various clinical parameters with expression level changes of these two GR isoforms. The obtained results showed no significant correlation between cGRα, cGR-P and cGRα/cGR-P mRNA expression and age, serum C-reactive protein (CRP) level, baseline serum total cortisol level, and white blood cell (WBC), red blood cell (RBC) and platelet (PLT) count of ill dogs at the time of admission to the hospital (Supplementary Fig. S4).

Since cGRα and cGR-P mRNA expression were varied in a wide range and displayed a high interindividual variability, we further subdivided SIRS patients into survivor and non-survivor groups. This kind of subdivision of the patients showed that baseline serum total cortisol was significantly higher (*p* = 0.0336) in the non-survivor than in the survivor group, medians were 320.0 nmol/l (41.6–1366.0 nmol/l) and 131.0 nmol/l (52.0–1131.0 nmol/l), respectively. The other characteristics—age, CRP level, and WBC, RBC and PLT count—did not significantly differ between the two groups (Supplementary Fig. S5).

**Figure 4:**
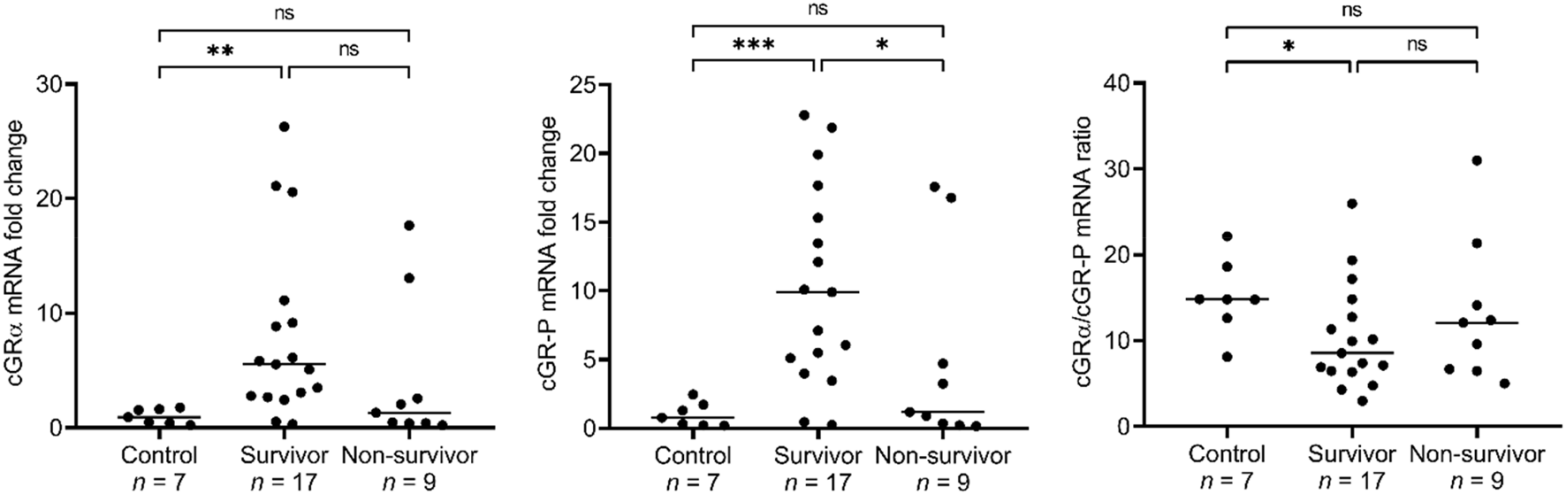
Expression of cGRα, cGR-P and ratio of cGRα/cGR-P mRNA in control, survivor and non-survivor SIRS patients. The expression of cGRα and cGR-P mRNA and their ratio in peripheral blood were compared between the control, survivor and non-survivor SIRS group. The number of dogs analysed in each group is indicated under the graphs. cGRα and cGR-P mRNA expressions were normalized to reference gene *RPS5* expression and presented as fold changes compared to the mean of the control group. Comparisons between two groups were performed by nonparametric Mann–Whitney *U* test. The horizontal lines represent the median values. Statistically significant differences are depicted by *p < 0.05, **p < 0.01, ***p < 0.001, *ns* non-significant.

The median mRNA expression of cGRα was increased 5.6-fold (0.3–26.3-fold) in survivor dogs with SIRS compared to healthy controls (*p* = 0.0008), whereas its expression did not differ significantly between the control and non-survivor group (*p* = 0.5360) (Fig. 4). The median mRNA expression of the cGR-P splice variant was increased 9.8-fold (0.3–22.8-fold) in survivors compared to controls (*p* = 0.0005), and similarly to cGRα expression, cGR-P expression in the non-survivor cohort did not significantly differ from that of the healthy controls (*p* = 0.3510) (Fig. 4). Interestingly, the ratio of median cGRα/cGR-P mRNA expression was not significantly different when the non-survivor cohort was compared to either survivor (*p* = 0.4337) or control group (*p* = 0.1738) (Fig. 4).

## Discussion

In this study, besides the full-length GRα, we identified an mRNA of intron 7 retention as the second most abundant splice variant of canine GR in the peripheral blood of a critically ill dog with septic peritonitis by RNA sequencing. The ubiquitous presence of this novel canine GR variant was corroborated by real-time PCR using isoform-specific primers and TaqMan probes in a cohort of healthy animals and dogs suffering from SIRS. This previously unknown canine splice variant encodes a truncated GR which lacks most of the ligand-binding domain of the wild-type protein. Amino acid sequence alignment of the truncated variant with human, pig and mouse GR-P isoforms demonstrated a high homology; thus, we designated it canine GR-P. Although our mRNA-Seq studies identified only two splice variants, it cannot be ruled out that there are more canine GR isoforms which are expressed in different tissues and/or diseases. However, according to human peripheral blood gene expression analysis the GRα and GR-P isoforms are the most abundant isoforms of the glucocorticoid receptor; therefore, we can assume that the shown expression data could represent the general GR expression in the whole blood of dogs.

The human GR-P transcript was initially identified in GC-resistant multiple myeloma and ACTH-producing small cell lung carcinoma cells and its excess was thought to contribute to the steroid resistance of these cells^14–17^. Since then, it has been reported that hGR-P mRNA is the second most abundant GR transcript of the transcriptional splice variants of hGRs in healthy patients; its amount could reach a quarter of those of the hGRα in peripheral blood mononuclear cells^9^. Analysis of porcine neuroendocrine tissues also presented a relatively high GR-P expression, 14–25% of total GR transcripts was given by this splice variant^23^. Investigation of GR-P mRNA amount in haematological malignancies^14,15,18,35–37^, tumors^16–18^, hyper- and hypocortisolism^9^, liver diseases^38^, critical illness^34,39^, kidney transplant patients^40^, psychiatric disorders^41,42^, and human placenta^43^ indicated an elevated level of expression of this isoform. The previously published GR-P mRNA levels are in agreement with our observation suggesting a generally high expression of this GR splice variant in mammals.

The cGR-P isoform identified by us is different from the previously reported cGRΔLBD splice variant that was found in a single dog with suspected iatrogenic Cushing’s syndrome^26^. The protein sequence of the cGRΔLBD and cGR-P isoforms differs in five amino acids only. The same research group expressed cGRΔLBD in COS-7 cells and found very low if any prednisolone-binding capacity of the truncated protein^26^ hinting that the cGR-P receptor described here is also unable to bind glucocorticoids. Negative GREs (nGRE) elements present in hundreds of genes coding for proinflammatory mediators are responsible for the GC-dependent repression of a wide array of target genes^44^. Proopiomelanocortin is an example of nGRE regulated genes and it was shown that though GR-P could bind to this nGRE, it failed to repress gene expression^45^. These findings suggest that GR-P preserves nGRE binding capacity but cannot recruit the transcription regulating proteins to the promoter. Surprisingly, a study aiming to reveal the physiological consequences of hGR-P protein expression demonstrated that transient coexpression of α and P isoforms augmented the hGRα response in a cell- and concentration-specific manner^18^. This result is bewildering considering that hGR-P and hGRβ—the other ligand-binding domain truncated GR isoform—have an inhibitory effect on transcriptional activation of glucocorticoid responsive genes and confer GC resistance^36^. The scarcely available and contradictory data call for further experiments to assign a more definitive physiological function to the GR-P protein.

According to certain studies expression of hGRα mRNA is not considerably different in the blood of patients with inflammatory diseases and healthy subjects^6,46^. However, the more generally accepted consensus states that inflammation is accompanied by elevated hGRα mRNA and protein expression and the lower baseline serum total cortisol level improves survival of the patients^34,47–49^. Furthermore, immune-mediated thrombocytopenia is accompanied by a significantly higher GRα mRNA level in glucocorticoid-responsive than in glucocorticoid-resistant patients^50^. We used cGRα- and cGR-P-specific probes to measure the GR transcript levels by real-time PCR in healthy and critically ill dogs with SIRS and demonstrated that mRNA expression of cGRα and cGR-P splice variant was increased in dogs with SIRS compared to healthy controls. Our results showing an increased expression level of the two most abundant isoforms of GR supplement the previously published results describing elevated mRNA level of GRα in inflammation.

To study their interdependence, we analysed and found a strong correlation between cGRα and cGR-P mRNA expression in critically ill dogs. A very few publications show simultaneous analysis of GRα and GR-P mRNA expression and most of the studies report similar GRα/GR-P ratios both in healthy subjects and patients of various diseases^9,18,37,39^. Thus, these results hint that expression of these two isoforms are collectively regulated.

Noteworthy, the GR expression data should be handled cautiously since the GRα and cGR-P mRNA expression has a high interindividual variability^10,29,35,51^. Nevertheless, most of the human data are in harmony with our results suggesting that simultaneous upregulation of GRα and GR-P mRNA expression attenuates inflammation and the ratio of these two isoforms is possibly not related to this effect.

The profound connection between cGRα and cGR-P mRNA expression and survival was clearly exposed when we divided the SIRS cohort into non-survivor and survivor patients. In comparison to healthy dogs, the expression of none of the two isoforms was elevated in the non-survivor patients while a significant mRNA level elevation of cGRα and cGR-P, 5.6- and 9.8-fold, respectively, was observed in samples of survivor dogs. Statistical analysis of cGRα/cGR-P mRNA ratios showed that there is no significant correlation between the ratio of the isoforms and survival.

Serum C-reactive protein is the major positive acute-phase protein in dogs and generally used as a biomarker of SIRS^32,52,53^. In our study, serum CRP-level did not discriminate dogs suffering from SIRS regarding survival; thus, serum CRP-level measured at hospital admission is not able to predict disease outcome in critically ill dogs^53,54^. According to our measurements, baseline serum total cortisol level varied in a wide range between survivor and non-survivor dogs but was significantly higher in the non-survivor group. Similar results were obtained by studying critically ill human and canine patients, i.e. plasma cortisol level varied in a wide range and mortality was associated with higher cortisol concentrations^46–49,55–58^. On the other hand, we could not find a significant relationship between the above-mentioned parameters such as serum CRP level and baseline serum total cortisol level and cGRα and cGR-P mRNA level or ratio of cGRα and cGR-P expression. These results show that the mRNA level of cGRα and cGR-P and baseline serum total cortisol concentration prognosticate the disease outcome in critically ill SIRS patients.

Critical illness results in activation of the HPA-axis, which might be accompanied by a peripheral adaptation in glucocorticoid sensitivity^39^. Increased expression of peripheral blood cell cGRα and cGR-P might be an adaptive mechanism for increased demand of the anti-inflammatory effect of cortisol. On the other hand, SIRS patients with markedly elevated serum cortisol level might have lower cGRα and cGRP expression due to the ligand-induced receptor downregulation^7^. Several human studies suggest that elevated serum cortisol level and/or decreased expression of GR in peripheral leukocytes correspond to a more severe disease course and poor outcome in patients with septic or non-septic SIRS^46–48^.

In conclusion, we described the previously unknown canine ortholog of GR-P and showed that this splice variant is the second most abundant GR mRNA in the peripheral blood of critically ill and healthy dogs. The level of cGRα and cGR-P transcripts are elevated in patients with systemic inflammatory response syndrome and the incidence of survival is higher in patients with increased cGRα and cGR-P mRNA expression. The relative expression of cGRα and cGR-P did not differ between the survivor and non-survivor patients suggesting that the total GR expression is more pertinent than the relative expression of GR isoforms. Considering that dog serves as a model species for several inflammatory conditions of human, e.g. sepsis^59^, rheumatoid arthritis^60^, atopic dermatitis^61^ and inflammatory bowel disease^62^, our studies could also contribute to the understanding of human glucocorticoid receptor signalling pathways.

## Methods

### Ethics declaration

This study involved critically ill dogs admitted to the Small Animal Hospital of the University of Veterinary Medicine (Budapest, Hungary) over a 4-year period (January 2014-March 2018). All dogs of this study were treated with a high standard of veterinary care. Written consent of the informed owner was also mandatory for inclusion. The study protocol was approved by the Animal Welfare Committee of the University of Veterinary Medicine (Budapest, Hungary) and was conducted in accordance with relevant guidelines and regulations. The authors complied with recommendations in the ARRIVE guideline. According to the Hungarian regulations on animal experimentation, our research does not qualify as an animal experiment.

### Collection of samples

Demographic data and selected clinicopathological data of the study subjects are summarized in Supplementary Table S1.

Twenty-six critically ill dogs with various inflammatory disorders were analysed in the study. The dogs classified according to SIRS criteria system^32^ with at least 2/4 changes were automatically enrolled in the study. When less than two criteria were changed or data were not available, inclusion into the study was based on individual evaluation of other clinicopathological data of patients. Haematological malignancies were exclusion criteria. Although the SIRS cohort was heterogeneous regarding the underlying cause and stage of the systemic inflammation, patients having pre-existing adrenal disease or receiving medications influencing the hypothalamic–pituitary–adrenal-axis (e.g. glucocorticoids, progestin, major analgesics, and azole antifungals) were excluded. All dogs underwent a complete physical examination, and venous blood sample was drawn after hospital admission. Complete Blood Count and serum biochemistry profile including serum CRP level (Canine CRP Diasystem Scandinavica) was measured. The limit of detection was 12.50 mg/l for serum CRP measurement and its value could not be measured in one ill dog due to insufficient sample volume, but this dog met 4 out of 4 criteria for SIRS. Baseline serum total cortisol level was detected by chemiluminescence immunoassay previously validated for canine serum sample (Immulite1000 Siemens). Two dogs with baseline serum total cortisol level exceeding the upper limit of quantification (1380 nmol/l) were excluded from the statistical analysis involving this parameter. Final diagnoses were concluded based on medical history, clinical signs, results of routine laboratory tests and diagnostic imaging, cytology/histopathology findings and/or pathology report. Dogs classified as survivors were alive when discharged from the ICU. Dogs were classified as non-survivors if died or were euthanized due to end-stage disease during the hospitalization period.

Seven enrolled healthy control subjects received no medication except regular treatment against endo- and ectoparasites. These dogs were considered healthy based on medical history and results of complete physical examination and blood tests.

Descriptive statistics of the demographic data and selected clinicopathological data of the subjects included in this study are presented in Supplementary Table S2.

### mRNA-Seq

mRNA-Seq was performed to identify splice variants of *NR3C1* gene from the peripheral blood sample (500 μl) of one critically ill dog with septic peritonitis secondary to foreign body ileus (Patient 25 in Supplementary Table S1). The sample was collected in Qiagen RNAprotect Animal Blood Tube (Qiagen) and the total RNA was extracted using RNeasy® Protect Animal Blood Kit (Qiagen) according to the manufacturer’s protocol. The RNA Integrity Number and RNA concentration were determined by RNA ScreenTape system with 2200 Tapestation (Agilent Technologies, Santa Clara, CA, USA) and RNA HS Assay Kit with Qubit 3.0 Fluorometer (Thermo Fisher Scientific, Waltham, MA, USA), respectively.

For mRNA-Seq library construction, KAPA Stranded mRNA-Seq Kit for Illumina (Roche) was applied according to the manufacturer’s protocol. The quality and quantity of the library was determined by using High Sensitivity DNA 1000 ScreenTape system with 2200 Tapestation (Agilent) and dsDNA HS Assay Kit with Qubit 3.0 Fluorometer (Thermo Fisher), respectively. Pooled libraries were diluted to 1.8 pM for 2 × 81 bp paired-end sequencing with 150-cycle High Output v2.5 Kit on the NextSeq 550 Sequencing System (Illumina) according to the manufacturer’s protocol.

The reads were adapter and quality trimmed (Q30) using built-in Illumina software (fastx-toolkit), and assessed for length, quality, adapter content, GC-content by FASTQC. The previously filtered, trimmed reads were mapped to Canis lupus Familiaris Assembly CanFam3.1 (Sep. 2011) by using HISAT2 alignment program (v2.1.0), then streamed into StringTie (v2.1.1) for transcript assembly without reference annotation guide to enable new transcript variant detection. Distribution of reads between different genomic regions (CDS exons, UTR exons, introns, upstream and downstream regions) were determined using RSeQC (v3.0.1). The resulting *NR3C1* transcripts were visually inspected using Integrative Genomics viewer, the consensus sequences of exons and presumed exons were concatenated and translated via EMBOSS transeq (v5.0.0).

The mRNA-Seq data have been deposited to the National Center for Biotechnology Information under BioProject ID PRJNA736937.

### Two-step RT-qPCR analysis

Peripheral blood RNA was collected and purified as described above. Concentration and purity of the isolated RNA were measured using NanoDrop spectrophotometer (Thermo Scientific) and 2100 Bioanalyzer (Agilent). In case of low A260/A230 ratios trace contaminants were removed by desalting method prior to cDNA synthesis. Integrity of isolated RNA was analysed by capillary electrophoresis and RNA extracts that passed the quality control were reverse transcribed to cDNA with RevertAid H Minus First Strand cDNA Synthesis Kit (Thermo Scientific) according to the manufacturer’s recommendations. Briefly, the genomic DNA contamination was removed by RNase-free DNase I treatment and the first strand of cDNA was synthesized by using a mixture of oligo(dT) and random hexamer primers.

Measurement of the dog cGRα and cGR-P mRNA expressions were performed with TaqMan-based real-time PCR assay in singleplex reactions (Supplementary Fig. S3). TaqMan primer sets and probes were designed with PrimerQuest™ Tool from IDT (available at https://eu.idtdna.com/) to amplify cGR isoforms. Primer specificity was checked against NCBI database using Primer-BLAST tool (available at https://www.ncbi.nlm.nih.gov/tools/primer-blast/).

The common forward primer (5’ GAT TAT TAA TGA GCA GAG AAT GAC CCT 3’) crossing exon 6–7 junction site that could anneal to both isoforms was used in conjunction with the reverse primers complementary to exon 8 (5’ GTT CCC TTC CCT CTT GAC AAT G 3’) and intron 7 (5’ GTT TCT GCA CAT TTA CAT TTC ATG C 3’) to amplify cGRα and cGR-P isoforms, respectively. The 5’ end HEX labelled cGRα-specific probe (5’ CAG TTC CTA AGG AAG GTT TGA AGA GCC A 3’) could bind to exon 7–8 junction site, while the 5’ end FAM tagged cGR-P-specific probe (5’ TGC TCT ACC AAC CTG AAG AGA GAA GC 3’) to the boundary of exon 7 and intron 7 (Supplementary Fig. S3). Both TaqMan probes were synthesized with BHQ1 quencher. The cGRα- and cGR-P-specific probes were designed on the antisense and sense strands, respectively.

The measurement was performed in a 10 μl final volume containing 1 μl cDNA, 1 × TaqMan™ Gene Expression Master Mix (Applied Biosystems), 900 nM primers (Eurofins Genomics AT) and 250 nM TaqMan probes (Eurofins Genomics AT).

Ribosomal protein S5 (*RPS5*) was used as the reference gene^63,64^ and its expression was measured in separate parallel assays by SYBR Green-based real-time PCR in 10 μl final volume containing 1 × qPCRBIO SyGreen Mix Lo-ROX reagent (PCR Biosystems), 1 μl of the reverse transcription reaction as template, 500 nM forward (5’ GGA GCG CCT GAC CAA CT 3’) and 500 nM reverse (5’ CCC GGG GAC CAC TGT TA 3’) primers. The primers were synthesized by IDT. Melting curve analysis and agarose gel electrophoresis of PCR products verified the specificity of the assay. The Ct values of the control and SIRS group samples did not differ significantly (Mann Whitney *U* test, *p* = 0.8228).

All real-time PCR reactions were carried out in MicroAmp® Fast 96-Well Reaction Plates (Applied Biosystems) in QuantStudio™ 12 K Flex Real-Time PCR System (Applied Biosystems) and the same PCR cycling parameters were used: 50 °C—2 min, 95 °C—10 min, followed by 40 cycles of 95 °C—15 s, 60 °C—1 min. Reactions were performed in triplicates and the primer and probe specificities were demonstrated by using no template and no reverse transcriptase negative controls. Serial dilutions of cDNA were measured to determine assay efficiencies^65^ according to the equation: PCR efficiency = (10[− 1/slope] − 1) × 100. The efficiencies were 95% (R^2^ = 0.9981), 80% (R^2^ = 0.9922) and 84% (R^2^ = 0.9916) for RPS5, cGR-α and cGR-P mRNA expressions, respectively.

All measured data was loaded into the relative quantitation analysis module on Thermo Fisher Cloud (Thermo Fisher Scientific) to export means ± SD of technical replicates. All technical replicates had a Ct standard deviation smaller than 0.3.

To calculate cGRα and cGR-P mRNA expression in control and SIRS samples, the 2^−ΔCt^ method was used^65^. Briefly, data were normalized to *RPS5* reference gene (listed in the last two columns of Supplementary Table S1) by using the formula 2^−ΔCt^, where ΔCt = Ct (target gene) – Ct (*RPS5* reference gene) of the sample. In the evaluation of survivor and non-survivor sub-cohorts these values were divided by the mean of the control group to indicate fold changes in mRNA expressions relative to the mean mRNA expression of healthy control dogs.

### In silico analysis

The sequences of NR3C1-201 (GRα) transcripts from different species were retrieved from Ensembl (available at http://www.ensembl.org/): human (ENST00000394464.7), porcine (ENSSSCT00000044530.2), murine (ENSMUST00000025300.13) and canine (ENSCAFT00000010207.4). The human, porcine, murine and canine exons were annotated based on genome assemblies GRCh38.p13, Sscrofa11.1, GRCm39 and CanFam3.1, respectively. The human sequence was used as reference to align the sequences and annotate the corresponding exons.

The amino acid sequence of human GRα (P04150-1) and GR-P (P04150-4) were obtained from UniProt Knowledgebase (available at https://www.uniprot.org/). The porcine, murine and canine GR-P isoform sequences were manipulated in Clone Manager 9 Professional Edition (v9.1). Porcine GR-P^23^ (accession number: KC329459) and partial murine GR-P^24^ (MG946717.1) transcript sequences were retrieved from NCBI. To construct the full murine GR-P coding sequence, the sequence from Ensembl was aligned to the partial GR-P sequence from GenBank and the intron 7 sequence was included after exon 7 of mouse GR. Canine GR-P transcript sequence was available from our mRNA-Seq results (deposited to GenBank, accession number: MZ382869). Translation into protein sequences based on the identified ORFs was done by Clone Manager 9 Professional Edition (v9.1). The hGRα ligand-binding domain was mapped between amino acids 531–777 by using NCBI Conserved Domain Search^66^. The amino acid sequences of GR-P isoforms of several species were aligned to hGRα with Clustal Omega (v1.2.4)^67^ and the point of divergence was identified as the amino acid 674 of hGRα.

### Statistical analysis

GraphPad Prism 8.0.1 statistical program was used for statistical analysis of clinical variables and gene expression levels.

Categorical variables (sex, neuter status) were expressed as frequencies. Continuous variables (age, white blood cell count, serum CRP concentration and baseline serum total cortisol level) were reported as median values with ranges.

Two-group comparisons of categorical variables were performed using Fisher’s exact test and in the case of continuous variables non-parametric Mann–Whitney *U* test was used. Correlation analysis was performed with Spearman’s rank correlation test. All *p* values reported are two-tailed and a *p*-value below 0.05 was taken to be statistically significant.

## Supplementary Information

Supplementary Information.

## Data Availability

The mRNA-Seq data have been deposited to the National Center for Biotechnology Information under BioProject ID PRJNA736937. The canine GR-P mRNA sequence identified by mRNA-Seq was deposited to GenBank under accession number MZ382869. All other data generated or analysed during this study are included in this published article and its Supplementary Information.
