# Supplementary material for: Restrained expression of canine glucocorticoid receptor splice variants α and P prognosticates fatal disease outcome in SIRS
Source: Sci Rep. 2021 Dec 30;11:24505. doi: 10.1038/s41598-021-03451-0 (PMC8718537; doi:10.1038/s41598-021-03451-0)
Supplement: Supplementary file 1 — Supplementary Information. [file 41598_2021_3451_MOESM1_ESM.pdf]

Restrained expression of canine glucocorticoid receptor splice variants α and P prognosticates fatal disease outcome in SIRS

Brigitta Margit Kállai[1], Judit Csöndes[2,3], Gergely Kiss[4], Lilla Bodrogi[5], Zsolt Rónai[1], and Tamás Mészáros[1,*]

[1]Department of Molecular Biology, Faculty of Medicine, Semmelweis University, Budapest, H-1085, Hungary

[2]Department of Clinical Pathology and Oncology, University of Veterinary Medicine, Budapest, H-1078, Hungary

[3]at the time of writing the manuscript, PraxisLab Ltd, Budapest, H-1038, Hungary

[4]Department and Clinic of Internal Medicine, University of Veterinary Medicine, Budapest, H-1078, Hungary

[5]Department of Animal Biotechnology, Institute of Genetics and Biotechnology, Hungarian University of Agriculture and Life Sciences, Gödöllő, H-2100, Hungary

*tamas.meszaros.su@gmail.com

Supplementary Figure S1. Nucleotide sequence alignment of the canine intron 8 sequence from Ensembl (upper) and mRNA-Seq (lower). Differences are depicted by orange highlights. Due to low coverage some parts of the sequence could not be read thus those bases are denoted by 'N'. Sequence alignment was performed by Clone Manager 9 Professional Edition.

```
1    GTTGGTAGAGCAACACTCTTTGCTTCTAAAATTCACTTCTAAGTACATAAAGAGCATGAA
1    ................................................C...........

61   ATGTAAATGTGCAGAAACAAAGGTTTTTTTTTTTTTTTTATACGTGGCCCCAAGTCTACC
61   .......C.....................G..............................

121  ATTATTATTTCCTTCTAGTCAGATATTTAGATTTTTCGTGAATGTCAAGTGTTATAAAGG
121  ............................................................

181  CACAGCTAGTGGTACCAAGATTAGTAAGACAGGTGCCATAGAAGAAGAGAGGAACATGAG
181  ...........................................A................

241  CTCTGCAGGAGCCTCTGACGATCTGGAGTGAAAACATGCTTCTTCCTCCCTGGGACCTTT
241  ............................................................

301  GGTAATAGCACCAGGGGAAAACTGGAGAAATAGGGCCAACCAGAATGGCCTCTGTTTCTC
301  ............................................................

361  TACTACATTGTAACTTGGTCAAAAATTAGTTCCCCAAGTATTATTACCATGTCCAGAAAT
361  ............................................................

421  GATGTGAGAAAAATAGGTCGTACGGAAGATCTACGTGATTACTACTAGAGACTTGGCTGA
421  ....................................................C.......

481  TGCAGCATTGCTGCTGCCATGGTGAATAGAGGTGTTTACCTAAAAAACAGCAACAATCTT
481  ........................G...................................

541  AAAAGTTTAGATAAAGCTACGGGTATATCAACTCCAGGGAGGTACGTTGTGGCTAGCAAA
541  ............................................................

601  GGCATTCCTTACTAAACCTAGAAAGCTTTTTGAAAAATACAAAGTCTCTTGGCAGTAAAA
601  ............................................................

661  AGGAGGCACCTTCAACAACCAGTACAAGAAGGTCTGTGAACAGTTGTTATACACATATAG
661  ............................................................

721  GCTATTCCAATAAAAATGACGTATCAGGTTAATGTTCATAAAATACTAAGCTGATGACTG
721  ............................................................

781  AATATTTCTTTCATATGCAAATCAATAAATTTGCAGTGTTTTAAAGTCTTCTTTCCCTGA
781  ............NNNNNNNNNNNNNNNNNNNNNNNNNNNNNNNNNNNNNNNNNNNNNNNN

841  ATAACTCTGGTTTCAGAACTGCTAGATAACTTTAAGCAACTGGATCTATTTCAGCTTTCC
841  NNNNN.......................................................

901  TAAGGCACACGTATCATTAAGCTTTCATATTACCTTTGTTCTACAGAAACCCTAAAGGAA
901  ............................................................

961  AAGATCACTGCTAGAGACTTTAAGGTTACCTAAGGCAGCACACTTGGTAAAGAAGCGAGC
961  ............................................................

1021 TCAATGTGATTTAACAGTTAAAATTCTGATAATGCCCAGATACAGATCAGTGCAAAATAC
1021 ............................................................

1081 TAACCTATCTTACTTGTCAAAAATGGTAATTTAAGTTGTATACATACCAGACTCACTAGC
1081 ............................................................

1141 TATATGTACTTTTTTTCATTGCATAGGATATAAAAAAGTAAACTGAACTACTATGTATCAC
1141 ............................................................

1201 TGGGTCCTTTACAAAAAAAAAAAAAAAGGATAGAATTAACAAGAAATAAATATATACCATA
1201 ............................................................
```

```
1261 AGATATAATCAGACTTCTACTCTGATGGAATATTAGAATAGATACCCTAACCTCCCACTA
1261 ............................................................

1321 AAAATGCTAGAGGAAGCAGGCAAAAAAACCAAAGAGGTTAGCTATACTAAGAGGGTATAT
1321 .......................................................C....

1381 TTAATAAGTTCAAAGACAGGAAACATACATGAAATTTTTAGTCAGAATGCAGTTTTTTTA
1381 ............................................................

1441 AATGCATATATAGGTACATGTGTGTACCATGTACATAAATGAACAGAATAAGAGATACGA
1441 ............................................................

1501 GTGAGGGAAGAGGGCTGAAACCGTGCTTTAAAAAAGTGATCCCATTTGTGACCACCATCA
1501 ............................................................

1561 CAAATGATTATCCTAACCCATTAGTTAGGATAACTATTAGTAACTACATTCATACATACT
1561 ............................................................

1621 AACTGCTATTAGTAACTACATTCACAGTATACCAGTTGAAAAAAAAGGCTTTGGTCTAAC
1621 ............................................................

1681 GTGATCATCAGAGATCCACAATGAAAGGTGGACAAGAATAGGGAAAGGAATTATACAAAG
1681 ............................................................

1741 AGTGGCTGAGAATTTTCTGGAATTGTGTAAAGACACCAACCCCCCCTACCTTACTAGGCA
1741 ............................................................

1801 GGCTCAGTGAGTTCCATATAACAACACAAGTAAAATCCAAATGACTGAAAAAAAAATCTT
1801 ............................................................

1861 CAAAGGAGAAGCAATTTAATAACCACAATAGCAAAGAAGAAAATGGAATGCTATATAACG
1861 ............................................................

1921 CTAAGGAAAAATATACTGGAAAATAAAGGTGGACTTCCAGTTTTAGCTCTGACAAACTGC
1921 ...............................A............................

1981 TTGGACAGTGCCATTCCCATTCTCCACAAGCAGCAGCTCAAATGAAAATCTGTAACTTTC
1981 ............................................................

2041 CTCAGATCTATCAGAGAATTGAGGTCACGAGGCAAATGACCACATCAGAATCTGGAGAAA
2041 ...G....................................N...................

2101 TAGGCAAACACAGAGACACACAGCCATGATCAGTTTATTAGGGACAGAAGAATCTGGAGC
2101 ............................................................

2161 CAGGAACTACTAGAAATGCTTACCGTAATTTTGATTAACTACTGAACAGGGAGGAAGAGC
2161 ............................................................

2221 TTGAAAATTCAAAACTAAGAGGGTTGTCTCCTCACAATTTCTTGGGGTTTACTCCAAGGC
2221 ................NN..................C.......................

2281 ATAAGCCACAACAGGTTCTCACCAGGAAGATCAGAGAAAAAGCCCCTGTTTTTTGCTCTC
2281 ............................................................

2341 TGAGGGAAGCTGCTGTGAGAGCTTCATCTGACCTGAGGGCAAGGGCAAAAAGATCATTGT
2341 ............................................................

2401 GGCCACCTCTCCCCTCCTCTCTCATGTAAGGGAAGCAGGAGTGTGAGGTGGGGGAGGAGG
2401 ............................................................

2461 TTCAGAAACACTTCTGAAGGTCATGACAAAACAGGTTTTATCATAAAAGGTAAGACACCC
2461 ............................................................

2521 CACATCCCCAGCACCTTACCACTGTATCAGTAGGGCTCCAGGATAGGAACTGGATTACAC
2521 .....................................................C......
```

```
2581 CTGCCAGAACTACAGAGCATGGCCTTTTGAGAAGTTCTCAGGGAAACCCAAAAACAACAG
2581 ............................................................

2641 GGGAAAAACAAAACAGGGAAACAGGTTTCAGTTATGAAACACAGCCCAGCTCTAGCCAGT
2641 ............................................................

2701 TTAACATAAGACCTTATACCAAAAGCATCATTATCTCAGCTCTGCTCTAGCACTATGCAG
2701 .................................................T..........

2761 CTTTCAGGGAGAAAAAAAAAAAAAAAAAGTAACGTATGCAGTAAGAATCTGAAGAGACAGT
2761 .............................................C..............

2821 CAAGCATCGGACCCAGCTCATCTATGACACAGATGTTTATCAGATAGGGAAGTGAACACA
2821 ............................................................

2881 ACCATGATGACTATGTTAAGGGCCCTAATGGAAAAAGTAAACAACATGCAAGAACACATG
2881 ............................................................

2941 AGTAATGTAAGCAGAGAGGTGGAAACTCTTAAGAATCAAAAGGAAATGCTAGAGACTAAA
2941 ............................................................

3001 AATACTGTAATGAAAATGGAGGATGGCCATCAGTAGACCAGACCTGGTGGAAACCCAAAC
3001 .........................................T..................

3061 TCAAGTGCAAAGAGAAGGATAAAAACAACATGGAACCGAGCATTCAAGAATGGTGAGGGC
3061 ............................................................

3121 TATTATACAATGTGTCCCGTGCAAGTAACGGGGACAGAAGTGGAAAAAAAAAAAAAAAAA
3121 ............................................................

3181 AAAAAGAATGGAAGAAATATATGAAGTAATAATGGCTGAGGGTTTTCCAAAATTAATGAC
3181 ............................................................

3241 GGATGCCAAAACATAGACCTAAACAGCTCAAAAAACTCCAAGAAAGATAAAAATATCTAC
3241 ............................................................

3301 ATCTAGGCATATTTTACTCAAACTGCAGAAAACCAAAGACAAGCAATCCTCAAAGAAACC
3301 ............................................................

3361 AGATGGAAAAAGAATCCGCTTTCTCATAGAGGAACAAAGATAAAAATGACATCAGAATGT
3361 ................A...........................................

3421 TCTCCACATGTAGGAAGGGAGTCAGGTGAAGCATTTAACGTTAAAAAAATATATATATCA
3421 ............................................................

3481 ATCTACAATTGGGTATTCAGTGAAATTGTCCTTCAAAAGTGAGGGTAAAATAAACAGTCT
3481 ............................................................

3541 CAAACAAAAACAGAATTAGTCACTAGAAGACCTGTCTTGCATGAAATGTTTAAGGAAGTT
3541 ............................................................

3601 CAGAGGAAATGAAAATGATGCAGTTCAGAAATTTGGATCTACATAAAGGAGAACATGAGA
3601 ................................G...........................

3661 GGAGGAAGCGATGAAAATGAAAATTGACTTTTCTTCTTACTTGATCTACCACACAACTGT
3661 ............................................................

3721 GGAAGGTGATTACAGCACCAGTACATCATAACAACAGGAGAGTGGACCCGGGCATACTGT
3721 ..........................................C.................

3781 TAGCGGGTACCTGCACTATATGGATCAAGTAGAATGGTCATATTTAAAGGAGCTGTAGAT
3781 ............................................................

3841 CCAGTGTCTATGTATATGTGGAACTCCAGGGCAAGCACTGAAACCACTTTAAATACACGT
3841 ............................................................
```

```
3901 AATAATTGATATCCTGAGAACAGAGAATATAGAATCATATGAAATGCTCATCAAAAACTG
3901 ............................................................

3961 GAGAAGTAGAAAAGAGGGAAGGATTACAAAAGGAAGCATCAGTGCAAAGAGTGCAAAACG
3961 ............................................................

4021 GTTACAAACATAGTACCTATTAACCCAAATATATCGAATGGTCACCTGACATGTGTATAG
4021 ............................................................

4081 TCAAAAAGTCTTTATTTTTATTTTTATTTTTATTTTTATTTTTTTTTAATGAAGGTGAAA
4081 ..........................A....T....N.......................

4141 GGAAAAGTTTGCCACCACCGATCCTCAATATAATACAAGACTAAAGGGTCTACTTAGGCT
4141 ............................................................

4201 AAGGGTAAGGAATCCCATGTCTAAGAAGTAAAGAGGAACAGAGAGAATGGTAATATGTAA
4201 ............................................................

4261 ATATGTTTAAGCTGACATCTATTATATAAAGTAACAGCAGTAGCGTTTTGGAAGATCTAA
4261 ............................................................

4321 AACCAGAACGCTAGAAGGATAGTTCAATAAGGAAGGTTCAAATTTCAATGACAATGGCTA
4321 ............................................................

4381 AAGATAGGAAAGGTACGCAGAGTACCAAAGCACCGAGGTGGGGGGGTGGAGCCCTTACGG
4381 ...........................................NNNNNNNNNNNNNNNNN

4441 AAAACACTGTAAGGAAAAGGTGGGGGGTGGAGCCCTTAAGGAAAACACTGTAAGGAAAA
4441 NNNNNNNNNNNNNNNNN...........................................

4501 GCAACCCAAACACATGAAATGAAGGGAAGGGGGTAAACAAGCACACAGGAAGGGAAAAAA
4501 ............................................................

4561 ACAAATGGTGAACAAATAAATTTATCTGCAATTTCATTAAATAGAGAGGAGATAAAAGTT
4561 ............................................................

4621 TCAACGTCAAAAGATACACAAAGAAATCCAAGACTGGATAAAAAAATAAAAAAAAAAAAA
4621 ............................................................

4681 GCTCCTTCCATGATACTGAATGATTGACATTAAAGGCCCTCCAGTAACCCAGTGGCGGTG
4681 ............................................................

4741 GACTTGTGCTGTAGAGCAGCATGTGGTAAATGTTCTCAGTGTTGCAAGCAAGACTGTCAC
4741 ............................................................

4801 AGCTGCTCAACTCTGCTCCTTGCAGCATGAACACAGTCATCACCTAAAGAAAGAACAGGC
4801 ............................................C...............

4861 ATGGTTATGTTCCAATAAAACTTTATTTACAAACACAGGCTGCTGGTGTGACCAGGCCAC
4861 ............................................................

4921 CAGTCGTGTTTTGCTGAGTGTTCTTTGAAACCAGAAGAAAGCTGTGGCAGCTTTCTTCAG
4921 ............................................................

4981 TTATGATCAGCACAGATGTTAAAGAGCTGGGTTTTTTTTTTTCTCACTTTATTTAAAGTT
4981 ...............................GG...........................

5041 ACTCCTTACATAAAGCATTCTACTTACCAGAAAAGTAATTCTAAGTATGTCGCTTAAACG
5041 ............................................................

5101 CATAGAGCAAAAATTAATGAAGCTCCAAAGATAATTAGGGGTAATTTGGGATAGATGGAA
5101 ............................................................

5161 TTCTTTTAAATATAAGCACTGATAGGACTTTGCCAATCATTATTTTTAGAGTTCAGGTAA
5161 ............................................................
```

```
5221 TAAGCAGGAAAAAAATACAGCTCAACAATCGATGAAAGTATTAAATTTTAGTATTTAAGA
5221 ............................................................

5281 AATTAGCAATAATTAGGTTATCTGTAACATATTAATGTACTTTTAACATGGTAAGCTCTT
5281 ............................................................

5341 AGCACTTCTGATGAAGTAGACTTCATTTTCACAGTTAATACTGACATCTTAGAAGTGCCA
5341 ............................................................

5401 AGTGTTGCTTTATAGAATCTGAACGTTTTCATGTGTGTTCCTTGGTCTAACCCTATTCAA
5401 ............................................................

5461 AATCCAGGGAGAGTGGTACACCACATTAACAAAATGAAAGGTAAAAGTCATACAATCATC
5461 ............................................................

5521 TCAACAACAGGTGCAGAAAAAGCATCTAACAAAATTCAGTGTCTGTTCATGATGAAAACA
5521 ............................................................

5581 TTCAACAAAATGAGTACAGAGGGACATACCTCAGCATAATAGCCATATATGACAACCCAC
5581 ............................................................

5641 AGCTAACATCATACTTCATGGTGAAAAGCTGAAAGCTTTCTGTCTACAATCAGGAATAAA
5641 ...............C............................................

5701 ACAAGGCTATCCACTGTCATCACTTTTATTCACCACAGTATTAAGGTTCTAGCCAGAGCA
5701 ............................................................

5761 ATTAGACAAGAAATATAAAAGGCATTCAAATCTAAAAGTACTAAATAAAACTCTCTCGGC
5761 ............................................................

5821 AGATGCCATGATATTATATATAGAAAAATTTAAAGACTCCACAAAGAGACCGTTAAAAAC
5821 ..................................................T.........

5881 AAGTAAGTTCATAAGGATGCAGGATACAATATCGATATGCAAAAATCTGCGGCAGTTCGA
5881 ............................................................

5941 GACACTAATAATAAGAACTATCAGAGAAACTAAAAAATATAACTCCATTTACATTTGCCT
5941 ............................................................

6001 CAAAAATAATATCTAGGAATAAAATGTTTTTAAGGAGGTGAAAGACCCATGCACTGAAAA
6001 ............................................................

6061 CAGTAAGACACTAATGAGAGAAATTGAAGATGACAGAAATAAACGCAAAGATGTTCCATA
6061 ............................................................

6121 CTCATGGATTAGAAGAATATTCTTAAAATGTTGACATTTCCAAAAGCAACCCACAAATTA
6121 ............................................................

6181 AGTGCAATCCCTGTTACAATTCCAGTGGCATTTTTCACAAAAGAAATCCTAAATTCTGTA
6181 ............................................................

6241 TAGAACTCTAAAAGACCCAGAATAGCCTAAGATACCGCAACATTACAGTACCACGTCTAA
6241 ...................................T..................A.....

6301 CTATAAAGCTTTCTCACCGCAAAGAAAATGATCAGCAAAATGAAAAAGCAGCCTACAGCA
6301 ............................................................

6361 TGGGAGAAAATCCTTGCAAATCCTAATTGGCCAAAGGGCACATGAAAAGATGCTCATCAT
6361 ..............G.............................................

6421 TACTAGTCATTAACAAGGAAATGCAAATCAAAACCACATGAGATCCCACCTGACCTCTAT
6421 ............................................................

6481 TAGAATGTCTGTTACCAAAAGACAAGGAATAAGTGCTGGCGAAGGTGTGGAGAAGAGGGA
6481 ............................................................
```

```
6541 AGCTGTACTCACTGCAGGTGGGAATACAAATTGATGCAGCCACCGTGGAAACAATATGGG
6541 ...........................................T................

6601 TGTTCCTCAACAAAGTGAAACAGGACTACTGCACGACCCTGCAGTTCCACTTCTGGGTAT
6601 ............................................................

6661 GTGTCCACAGAAAATGAAAATACTCGAAAAATATATGTAGCCTCATACTCATGGCATAAT
6661 ............................................................

6721 TACTGATAGTAGCTAAGATACGGAAGCAGCTGAAGAGTCCACCTATGCAAGAGTCCACCT
6721 ............................................................

6781 ATGGTTTGTTTAGTAAATATATCCAATAGACCATTATTCCACCATAGAAAGGAGTGGAAT
6781 ............................................................

6841 CTTGCCACTTGCAGCAGTTTGGATGGACCTTGAAGGCACTGCGCTAAGTGAAATAAGCCA
6841 ..........................................A.................

6901 GTCAGAGAAAACTGAGCACAAACGAATATATATAGGGACGCCTGGGTGGCTCAGTGGTTG
6901 ............................................................

6961 AGCATCAGCCTTTGGCTTAGATTGTGATCCCGGGGTCCTGGGATCGAGTCTCACATCTGG
6961 ............................................................

7021 CTCCCTGTGAGGAACCTGCTTCTCCCTCTGCCTCTCTCTCGGTGTCTCTCATGAATAAAT
7021 ............................................................

7081 AAAATCTTAAAAAAAAAAATATATATAATCTCACTTATACGTGGACTCTAAAAATAAATA
7081 ...................AG.......................................

7141 GGGACACGGGCTCTTGTATGCAGAGAACAGACTGGCAGTCTCCAGCGGAGGGTGTTTTGG
7141 .............................................A..............

7201 AGGGTGGGAGAAATGAGGGAAGGAGAGGGTTAAAAAGTGCAAACTTCCCGGTATAAAATA
7201 ............................................................

7261 AGTCACATGGATGTCATGTGCAGCTTGGTGACTATAGTTATTAATAATATATATTATATA
7261 ............................................................

7321 TATGAAAGTTGCTCAGTAAATATTAAATGTCATCATGAGAAAAAAATGTATACTATCTGA
7321 ............................................................

7381 TGATGGATGTTAACTATAGATGTATTGTGATCGTTTTGCAATATATACAAATATTTGGTC
7381 ............................................................

7441 ATTCTGTTGTACAACCTGAAACTAATGTCAATTTTATCTAAATTTTTAGAACTTAAAACT
7441 ............................................................

7501 CCATGAGAAAGATTAGGACATGCAGCTAAGGCAGGTTATACTCAAATCCACTGGACAAGT
7501 ............................................................

7561 AAGAATTAAATCTTCCTGCTCCTAAAACCACTGCTTTTCCTTACCTTAATTCCAGCATTT
7561 ............................................................

7621 TCTAAGATGTCATCAACAATGAACTTAAGTTTGGGGAAACATCATGTTCCCTTCCCTAAT
7621 ............................................................

7681 TCTGGCTCTGATAAATCCTGAAAACCAATAGTCTACCTAACTTTGTATTAACCTTTAATT
7681 ............................................................

7741 TTGCTTACATTTTGACCAAATCCTTTTTTCCCTAACCATTAACACAGAACCAAAGTTCTG
7741 ............................................................

7801 AGGAGTACACTTGGAGAAATTTGGCCATGTACAAAAATCAGTCAGTCTTTGCAGGATTCA
7801 ............................................................
```

7861 TGAACATGATTAACCTTCTGTTCACAGTCCTTCTTACTGGTATCAGTTTTTGAAGAGTAA
7861 ....

7921 TAAATGATTCATTTTTATACCGATTTTACAATCTGGAATCCATAAGTTTTTTTCTAAGTG
7921 ....

7981 CGTAAGGGAAGGCTGATACATGCTCCCCAGCAAGGCAAGACTCCAGGTTAAATCCTCTGA
7981 ....

8041 TTTTCTATTATCCCCTCATCTCTCTTGCCTGTACTTTCAGGATCCTGTGAGATGCTTGTA
8041 ....

8101 TTTTCATGGAATGTATCTAGAGTGTATATACAATACATGTCATGAAGCATGCTATTGTTA
8101 ....

8161 ATAAATGCCCTACAGGCACATAGGCAGAGTTACCTTAAGAAAATTCTTTCCTTGACCACA
8161 ....

8221 TCTTAACCTTTTAG
8221 ....

Supplementary Figure S2. Amino acid sequence alignment of human (h), porcine (p), murine (m) and canine (c) GR-P isoforms to hGRα. Sequence alignment was performed by Clustal Omega (v1.2.4). Fully conserved residues are depicted with asterisks, strongly and weakly similar residues with colons and periods, respectively. Numbers at the end of the rows refer to amino acid positions.

```
hGRα      MDSKESLTP-GREENPSSVLAQERGDVMDFYKTLRGGATVKVSASSPSLAVASQSDSKQR   59
hGR-P     MDSKESLTP-GREENPSSVLAQERGDVMDFYKTLRGGATVKVSASSPSLAVASQSDSKQR   59
pGR-P     MDPKESLTPPSREEIPSSVLGRERAHVMDFYKSLRGGTPVKVSAASPSLAAVSQPDSKQQ   60
mGR-P     MDSKESLAPPGRDEVPSSLLGRGRGSVMDLYKTLRGGATVKVSASSPSVAAASQADSKQQ   60
cGR-P     MDSKESLSPPSKEEISSSVLGRERGNVMDFYKTLRGGATVKVSASSPSLAAASQSDSKQQ   60
          ** ****:* .::*  **:*.: *. ***:**:****: *****:***:*..** ****:

hGRα      RLLVDFPKGSVSNAQ--------QPDLSKAVSLSMGLYMGETETKVMGNDLGFPQQGQIS   111
hGR-P     RLLVDFPKGSVSNAQ--------QPDLSKAVSLSMGLYMGETETKVMGNDLGFPQQGQIS   111
pGR-P     RLAVDFPKGSGSNAQ--------QPDLSKAVSLSMGLYMGETETKVMGSDLGFPQQGQIS   112
mGR-P     RILLDFSKGSASNAQQQQQQQQPQPDLSKAVSLSMGLYMGETETKVMGNDLGYPQQGQLG   120
cGR-P     RLLVDFPKGSGSHAQ--------QPDLSKAVSLSMGLYMGETETKVMGNDLGFPQQGQIS   112
          *: :** *** *:**        *************************.***:*****:.

hGRα      LSSGETDLKLLEESIANLNRSTSVPENPKSSASTAVSAAPTEKEFPKTHSDVSSEQQHLK   171
hGR-P     LSSGETDLKLLEESIANLNRSTSVPENPKSSASTAVSAAPTEKEFPKTHSDVSSEQQHLK   171
pGR-P     LSSGETDFRLLEESIANLSRSTSVPENPKSSASAAGSAAPAEKAFPKTHSDGAPEQPNVK   172
mGR-P     LSSGETDFRLLEESIANLNRSTSRPENPKSSTPAAGCATPTEKEFPQTHSDPSSEQQNRK   180
cGR-P     LSSGETDFRLLEESIANLNRSTSVPENPKSSVS-AVSAAPTEKEFAQTLSDVSSEQQHLK   171
          *******::*********.**** *******.  * .*:*:** * :* ** : ** : *

hGRα      GQTGTNGGNVKLYTTDQSTFDI----LQDLEFSSGSPGKETNESPWRSDLLIDENCLLSP   227
hGR-P     GQTGTNGGNVKLYTTDQSTFDI----LQDLEFSSGSPGKETNESPWRSDLLIDENCLLSP   227
pGR-P     GQTGTNGGNVKLFTTDQSTFDIWRKKLQDLELPSGSPGKETSESPWSSDLLIDENCLLSP   232
mGR-P     SQPGTNGGSVKLYTTDQSTFDI----LQDLEFSAGSPGKETNESPWRSDLLIDEN-LLSP   235
cGR-P     GQTGTNGGNVKLYTTDQSTFDVWRKKLQDLDFTSGSPEKEASESPWRSDLLIDENCLLSP   231
          .* *****.***:********:    ****:: :*** **:.**** ******** ****

hGRα      LAGEDDSFLLEGNSNEDCKPLILPDTKPKIKDNGDLVLSSPSNVTLPQVKTEKEDFIELC   287
hGR-P     LAGEDDSFLLEGNSNEDCKPLILPDTKPKIKDNGDLVLSSPSNVTLPQVKTEKEDFIELC   287
pGR-P     LAGEEDPFLLEGSSTEDCKPLVLPDTKPKVKDNGELILPSPNSVPLPQVKTEKEDFIELC   292
mGR-P     LAGEDDPFLLEGDVNEDCKPLILPDTKPKIQDTGDTILSSPSSVALPQVKTEKDDFIELC   295
cGR-P     LAGEDDPFLLEGNSNEDCKPLVLPDTKPKIKDNGDLILASPNSVPLPQVKTEKEDFIELC   291
          ****:* *****. .******:*******::*.*: :* **..* ********:******

hGRα      TPGVIKQEKLGTVYCQASFPGANIIGNKMSAISVHGVSTSGGQMYHYDMNTA-SLSQQQD   346
hGR-P     TPGVIKQEKLGTVYCQASFPGANIIGNKMSAISVHGVSTSGGQMYHYDMNTA-SLSQQQD   346
pGR-P     TPGVIKQEKLGPAYCQASFSGANIIGGKMSAISVHGVSTSGGQLYHYDMNTAASLSKQQE   352
mGR-P     TPGVIKQEKLGPVYCQASFSGTNIIGNKMSAISVHGVSTSGGQMYHYDMNTA-SLSQQQD   354
cGR-P     TPGVIKQEKLGPVYCQASFSGANIIGNKMSAISVHGVSTSGGQMYHYDMNTA-SLSQQQD   350
          *********** .****** *:****.****************:******** ***:**:

hGRα      QKPIFNVIPPIPVGSENWNRCQGSGDDNLTSLGTLNFPGRTVFSNGYSSPSMRPDVSSPP   406
hGR-P     QKPIFNVIPPIPVGSENWNRCQGSGDDNLTSLGTLNFPGRTVFSNGYSSPSMRPDVSSPP   406
pGR-P     QKPLFNVIPPIPVGSENWNRCQGSGDDNLTSLGTLNFSGRSVFSNGYSSPGMRPDVSSPP   412
mGR-P     QKPVFNVIPPIPVGSENWNRCQGSGEDNLTSLGAMNFAGRSVFSNGYSSPGMRPDVSSPP   414
cGR-P     QKPIFNVIPPIPVGSENWNRCQGSGDDNLASLGTLNFPGRSVFSNGYSSPGMRPDVSSPP   410
          ***:*********************:***:***::** **:*********.*********

hGRα      SSSSTATTGPPPKLCLVCSDEASGCHYGVLTCGSCKVFFKRAVEG-QHNYLCAGRNDCII   465
hGR-P     SSSSTATTGPPPKLCLVCSDEASGCHYGVLTCGSCKVFFKRAVEG-QHNYLCAGRNDCII   465
pGR-P     SSSS-AATGPPPKLCLVCSDEASGCHYGVLTCGSCKVFFKRAVEG-QHNYLCAGRNDCII   470
mGR-P     SSSS-TATGPPPKLCLVCSDEASGCHYGVLTCGSCKVFFKRAVEG-QHNYLCAGRNDCII   472
cGR-P     SSSS-AATGPPPKLCLVCSDEASGCHYGVLTCGSCKVFFKRAVEGRQHNYLCAGRNDCII   469
          **** ::************************************** **************

hGRα      DKIRRKNCPACRYRKCLQAGMNLEARKTKKKIKGIQQATTGVSQETSENPGNKTIVPATL   525
hGR-P     DKIRRKNCPACRYRKCLQAGMNLEARKTKKKIKGIQQATTGVSQETSENPGNKTIVPATL   525
pGR-P     DKIRRKNCPACRYRKCLQAGMNLEARKTKKKIKGIQQATTGVSQETSENSANKTIVPATL   530
mGR-P     DKIRRKNCPACRYRKCLQAGMNLEARKTKKKIKGIQQATAGVSQDTSEN-ANKTIVPAAL   531
cGR-P     DKIRRKNCPACRYRKCLQAGMNLEARKTKKKIKGIQQATPGASQETSENPANKTIVPATL   529
          *************************************** *.**:**** .*******:*
```

```
hGRα     PQLTPTLVSLLEVIEPEVLYAGYDSSVPDSTWRIMTTLNMLGGRQVIAAVKWAKAIPGFR    585
hGR-P    PQLTPTLVSLLEVIEPEVLYAGYDSSVPDSTWRIMTTLNMLGGRQVIAAVKWAKAIPGFR    585
pGR-P    PQLTPTLVSLLEVIEPEVLYAGYDSSIPDSTWRIMTALNMLGGRQVIAAVKWAKAIPGFR    590
mGR-P    PQLTPTLVSLLEVIEPEVLYAGYDSSVPDSAWRIMTTLNMLGGRQVIAAVKWAKAIPGFR    591
cGR-P    PQLTPTLVSLLEVIEPEVLYAGYDSSVPDSTWRIMTTLNMLGGRQVIAAVKWAKAIPGFR    589
         **************************:***:*****:***********************

hGRα     NLHLDDQMTLLQYSWMFLMAFALGWRSYRQSSANLLCFAPDLIINEQRMTLPCMYDQCKH    645
hGR-P    NLHLDDQMTLLQYSWMFLMAFALGWRSYRQSSANLLCFAPDLIINEQRMTLPCMYDQCKH    645
pGR-P    NLHLDDQMTLLQYSWMFLMAFALGWRSYRQSSASLLCFAPDLVINEQRMALPCMYDQCRH    650
mGR-P    NLHLDDQMTLLQYSWMFLMAFALGWRSYRQASGNLLCFAPDLIINEQRMTLPCMYDQCKH    651
cGR-P    NLHLDDQMTLLQYSWMFLMAFALGWRSYRQSSGSMLCFAPDLIINEQRMTLPCMYDQCKH    649
         ******************************:*..:*******:******:********:*

hGRα     MLYVSSELHRLQVSYEEYLCMKTLLLLSSVPKDGLKSQELFDEIRMTYIKELGKAIVKRE    705
hGR-P    MLYVSSELHRLQVSYEEYLCMKTLLLLSSGW-----------------------------    676
pGR-P    MLYVSSELQRLQVSYEEYLCMKTLLLLSSGW-----------------------------    681
mGR-P    MLFISTELQRLQVSYEEYLCMKTLLLLSSGWWTTYSLLTFILN--------MANAC----    699
cGR-P    MLFVSSELQRLQVSYEEYLCMKTLLLLSSGW-----------------------------    680
         **::*:**:**********************

hGRα     GNSSQNWQRFYQLTKLLDSMHEVVENLLNYCFQTFLDKTMSIEFPEMLAEIITNQIPKYS    765
hGR-P    ------------------------------------------------------------    676
pGR-P    ------------------------------------------------------------    681
mGR-P    ----ESC---------ITIVHICVYICMCVCIYIYIYIYIYIYICQFHCVFLVR------    740
cGR-P    ------------------------------------------------------------    680

hGRα     NGNIKKLLFHQK    777
hGR-P    ------------    676
pGR-P    ------------    681
mGR-P    ------------    740
cGR-P    ------------    680
```

Supplementary Figure S3

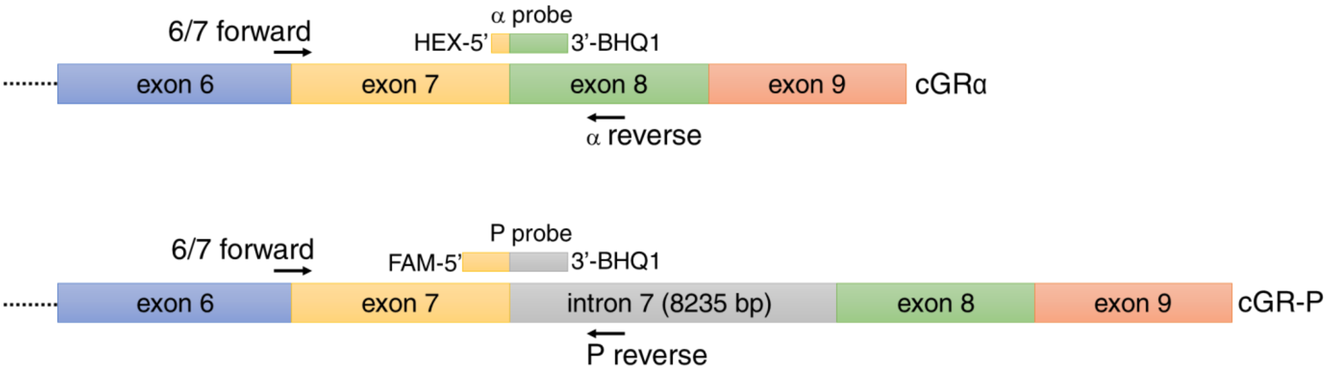

Supplementary Figure S3. Schematic drawing of the two identified glucocorticoid receptor splice isoforms and their specific real-time PCR primers and probes.

Supplementary Figure S4

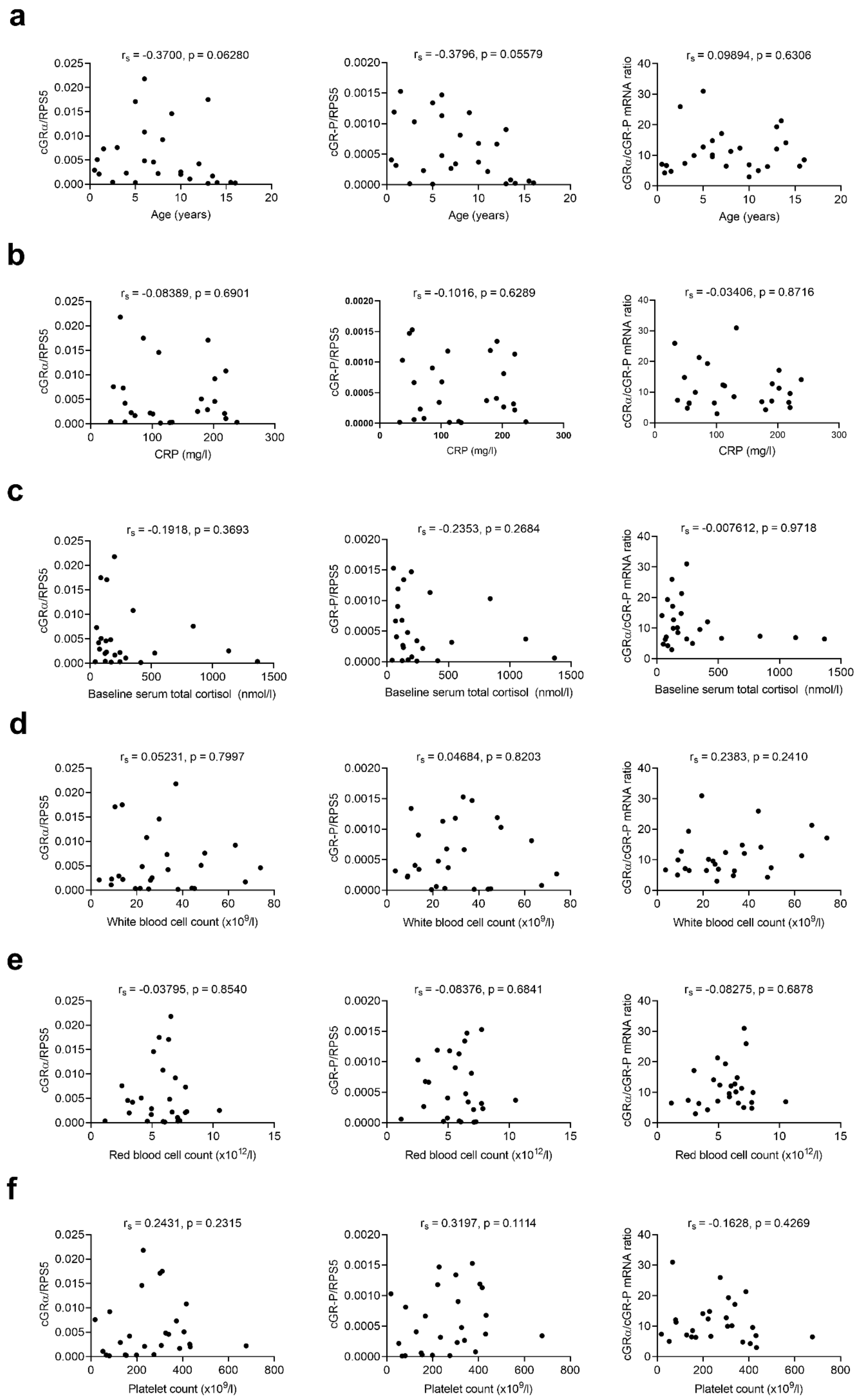

Supplementary Figure S4. Correlations between SIRS patient variables at hospital admission and cGRα, cGR-P, and ratio of cGRα/cGR-P mRNA expression. The investigated sample variables are **(a)** age (*n* = 26), **(b)** CRP level (*n* = 25), **(c)** baseline serum total cortisol level (*n* = 24), and **(d)** white blood cell (*n* = 26), **(e)** red blood cell (*n* = 26) and **(f)** platelet count (*n* = 26) with the sample size indicated between brackets. cGRα and cGR-P mRNA expressions are normalized to reference gene *RPS5* expression. Correlations were analysed by Spearman's rank test and $p < 0.05$ was considered significant.

Supplementary Figure S5

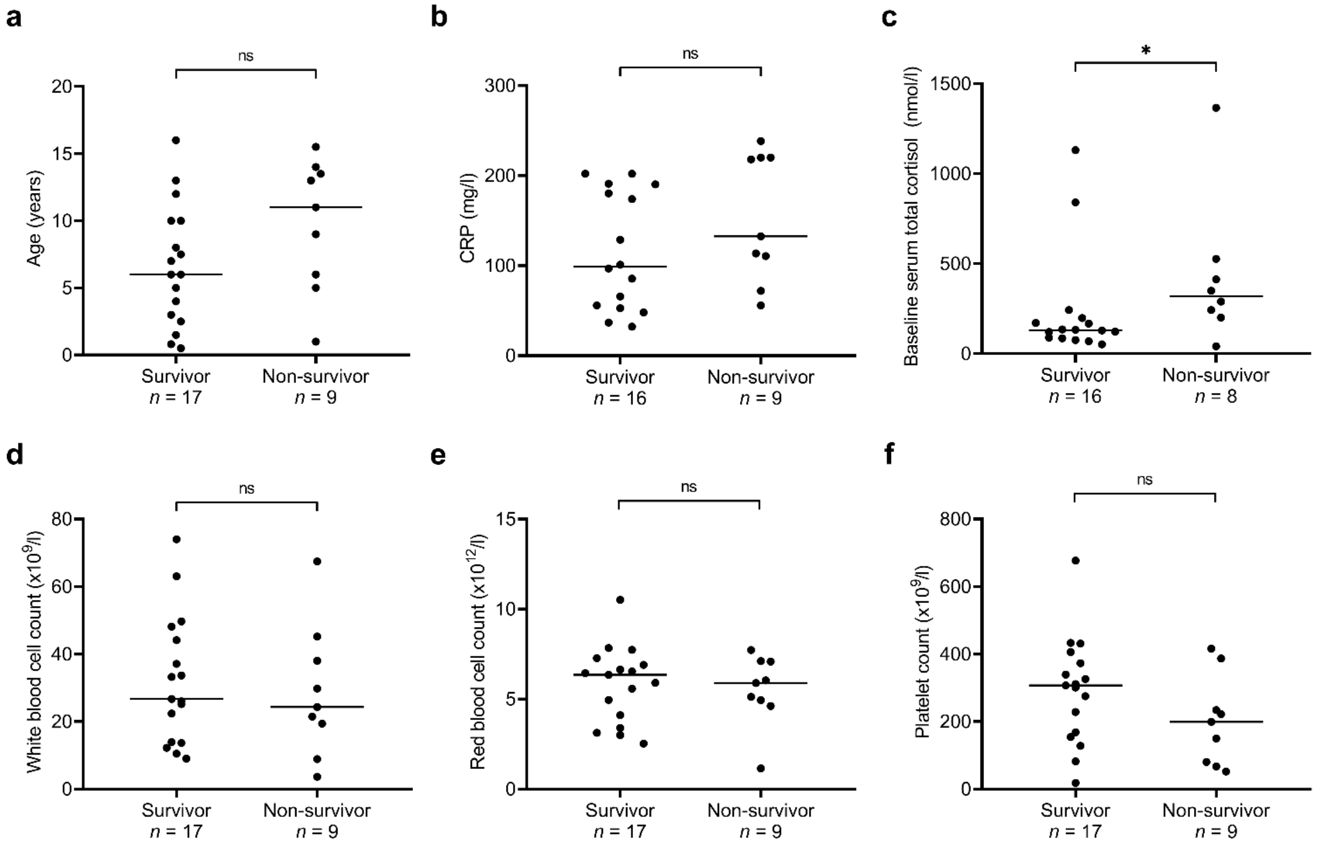

Supplementary Figure S5. Comparison of patient parameters of the survivor and non-survivor SIRS group. The investigated sample characteristics are **(a)** age, **(b)** CRP level **(c)** baseline serum total cortisol level, and **(d)** white blood cell, **(e)** red blood cell and **(f)** platelet count. The number of dogs studied in each case are indicated under the graphs. The lines represent the median values. Comparisons were performed by nonparametric Mann-Whitney *U* test. Statistically significant differences are depicted by *$p < 0.05$ and ns = non-significant.

Supplementary Table S1. Demographic data and selected clinicopathological data of the study subjects.

Patient number	Group	Breed	Sex	Neutered/ Spayed	Age (years)	White blood cell count (x10^{9}/l)	Red blood cell count (x10^{12}/l)	Platelet count (x10^{9}/l)	Serum C-reactive protein (mg/l)	Baseline serum total cortisol (nmol/l)	Diagnosis	Disease outcome	cGRα/RPS5	cGR-P/RPS5
Control1	control	Akita inu	male	no	2,2	8,03	6,51	127,00	12,5	168,0	-	-	0,001289	0,000087
Control2	control	Shiba inu	male	no	4,5	9,05	7,63	227,00	12,5	59,3	-	-	0,000769	0,000052
Control3	control	Labrador Retriever	female	yes	2,2	9,93	6,06	272,00	35,8	51,3	-	-	0,001341	0,000165
Control4	control	Mixed breed	female	yes	4,5	8,28	8,50	360,00	12,5	175,0	-	-	0,001462	0,000116
Control5	control	Mixed breed	male	yes	3,2	21,80	6,52	283,00	12,5	132,0	-	-	0,000207	0,000014
Control6	control	Mixed breed	female	yes	1,0	13,70	7,76	264,00	12,5	315,0	-	-	0,000337	0,000015
Control7	control	Mixed breed	female	yes	1,0	18,50	5,29	309,00	21,2	249,0	-	-	0,000400	0,000022
Patient1	SIRS	Bichon Havanese	male	yes	6,0	37,10	6,54	228,00	48,0	198,0	purulent septic pleuritis	survivor	0,021793	0,001471
Patient2	SIRS	Cocker Spaniel	female	yes	6,0	22,40	6,44	326,00	N/A	167,0	pleuropneumonia<br>heartworm infection<br>polyradiculoneuritis<br>cysta ovarii	survivor	0,004843	0,000477
Patient3	SIRS	Cavalier King Charles Spaniel	male	yes	3,0	49,70	2,53	18,00	36,8	841,0	diabetes mellitus<br>pancreatitis	survivor	0,007594	0,001029
Patient4	SIRS	English Cocker Spaniel	male	no	15,5	21,47	1,15	150,00	55,8	1366,0	immune-mediated haemolytic anaemia<br>multiple organ dysfunction syndrome	non-survivor	0,000392	0,000061
Patient5	SIRS	Great Dane	male	yes	1,5	33,21	7,74	373,00	52,8	52,0	aspiration pneumonia	survivor	0,007330	0,001532
Patient6	SIRS	German Shepherd Dog	male	no	11,0	8,88	7,08	52,00	220,0	290,0	neoplasma pulmonum<br>septicaemia	non-survivor	0,001093	0,000218
Patient7	SIRS	Golden Retriever	male	yes	13,0	13,67	5,58	311,00	85,5	86,0	aspiration pneumonia<br>megaoesophagus	survivor	0,017494	0,000904
Patient8	SIRS	Chinese Shar-Pei	female	no	9,0	29,80	5,12	222,00	110,7	1380,0	*Babesia canis* infection<br>urothelial cell carcinoma<br>hydronephrosis<br>pancreatitis<br>acute kidney injury<br>multiple organ dysfunction syndrome	non-survivor	0,014639	0,001182
Patient9	SIRS	Czechoslovakian Wolfdog	male	yes	1,0	3,63	7,73	234,00	218,0	527,0	pneumonia	non-survivor	0,002124	0,000317
Patient10	SIRS	Doberman Pinscher	female	no	6,0	24,33	5,89	416,00	220,0	350,0	mastitis	non-survivor	0,010821	0,001127
Patient11	SIRS	Wirehaired Dachshund	female	yes	7,0	74,00	3,00	339,00	202,0	129,0	pleuropneumonia	survivor	0,004604	0,000268
Patient12	SIRS	Pug	female	no	13,5	67,43	4,94	387,00	71,9	201,0	protein-losing enteropathy<br>lymphoplasmocytic gastroenteritis<br>hydrothorax<br>ascites<br>oedema pulmonum	non-survivor	0,001704	0,000080
Patient13	SIRS	Mixed breed	male	no	8,0	63,06	6,90	82,00	202,0	1380,0	abscess<br>endocarditis<br>thrombosis<br>multiple organ dysfunction syndrome	survivor	0,009214	0,000813
Patient14	SIRS	Bichon Havanese	male	no	5,0	10,50	6,35	301,00	191,0	135,0	bite wound<br>traumatic abdominal hernia<br>pneumothorax<br>purulent septic peritonitis	survivor	0,017063	0,001339
Patient15	SIRS	German Shepherd Dog	male	yes	0,5	12,20	4,95	128,00	190,2	76,0	bronchopneumonia	survivor	0,002901	0,000407

Patient number	Group	Breed	Sex	Neutered/ Spayed	Age (years)	White blood cell count ($x10^9/l$)	Red blood cell count ($x10^{12}/l$)	Platelet count ($x10^9/l$)	Serum C-reactive protein (mg/l)	Baseline serum total cortisol (nmol/l)	Diagnosis	Disease outcome	cGRα/RPS5	cGR-P/RPS5
Patient16	SIRS	Cane Corso - mixed breed	female	no	10,0	26,70	10,51	431,00	174,0	1131,0	acute gastroenteritis<br>multiple organ dysfunction syndrome	survivor	0,002556	0,000370
Patient17	SIRS	Bullterrier	male	no	12,0	33,67	3,40	168,00	55,8	69,0	purulent septic peritonitis<br>linear foreign body ileus and intestinal perforation	survivor	0,004219	0,000665
Patient18	SIRS	Landsheer	male	no	4,0	9,04	7,84	307,00	65,8	133,0	purulent peritonitis<br>pancreatitis	survivor	0,002313	0,000233
Patient19	SIRS	Mixed breed	male	yes	0,8	48,12	4,11	406,00	180,3	89,0	fever of unknown origin	survivor	0,005087	0,001187
Patient20	SIRS	Westhighland White Terrier	female	no	7,5	13,93	6,64	677,00	96,8	243,0	oesophagitis<br>oesophageal foreign body	survivor	0,002222	0,000343
Patient21	SIRS	Bichon Bolognese	female	yes	10,0	26,01	3,13	433,00	101,0	121,0	stump pyometra<br>peritonitis<br>immune-mediated haemolytic anaemia	survivor	0,002028	0,000678
Patient22	SIRS	Samoyed	male	no	16,0	25,20	5,90	154,00	128,8	171,0	pancreatitis<br>purulent peritonitis	survivor	0,000262	0,000031
Patient23	SIRS	Neapolitan Mastiff	female	yes	13,0	38,00	6,04	80,00	113,6	413,0	purulent septic peritonitis<br>foreign body ileus	non-survivor	0,000184	0,000015
Patient24	SIRS	Dachshund	male	no	5,0	19,40	7,11	67,00	132,5	243,0	pancreatitis<br>purulent peritonitis	non-survivor	0,000346	0,000011
Patient25	SIRS	Mixed breed	male	no	2,5	44,10	7,27	275,00	32,2	123,0	purulent septic peritonitis<br>foreign body ileus	survivor	0,000437	0,000017
Patient26	SIRS	Russian Black Terrier	male	no	14,0	45,20	4,61	199,00	238,3	41,6	endocarditis	non-survivor	0,000342	0,000024

N/A: not available

Supplementary Table S2. Descriptive statistics of the demographic data and selected clinicopathological data of the study subjects.

Variable	Control (*n* = 7)	SIRS (*n* = 26)	*p*-value
**Sex**			
female	4 of 7	9 of 26	0.3926[a]
male	3 of 7	17 of 26	
**Neutered or spayed**	5 of 7	11 of 26	0.2245[a]
**Age (years)**	2,2 (1,0-4,5)	7,3 (0,5-16,0)	0.0085[b]
**White blood cell count (x10^9/l)**	9,93 (8,03-21,80)	26,36 (3,63-74,00)	0.0056[b]
**Red blood cell count (x10^{12}/l)**	6,52 (5,29-8,50)	5,97 (1,15-10,51)	0.1197[b]
**Platelet count (x10^9/l)**	272,0 (127,0-360,0)	254,5 (18,0-677,0)	>0.9999[b]
**Serum C-reactive protein (mg/l)**	12,5 (12,5-35,8)	113,6 (32,2-238,3)	<0.0001[b]
**Baseline serum total cortisol (nmol/l)**	168 (51,3-315)	184,5 (41,6-1380.0)	0.4178[b]

Data are presented as frequencies or medians (range).

[a]Fisher's exact test

[b]Mann-Whitney *U* test

Supplementary Table S3. Summary of mRNA-Seq data obtained by analysing peripheral blood of a critically ill dog with septic peritonitis.

**Total Reads:**	44691037
**Total Tags:**	55127177
**Total Assigned Tags:**	51250379

Group		Total_bases	Tag_count	Tags/Kb
**CDS_Exons**		33014650	28742753	870.61
**5'UTR_Exons**		7221338	838597	116.13
**3'UTR_Exons**		19511230	5396171	276.57
**Introns**		917539223	9911519	10.8
**Transcription Start Site upstream**	**1 kb**	24856465	290963	11.71
	**5 kb**	112512194	751922	6.68
	**10 kb**	204774660	1092330	5.33
**Transcription End Site**	**1 kb**	25081957	2448377	97.62
	**5 kb**	110516934	4789196	43.33
	**10 kb**	197949325	5269009	26.62
***NR3C1* exons**		7268	6140	844.80
***NR3C1* 7-8 intron**		8234	1277	155.09

**Global intron-exon ratio**	0.012405095
***NR3C1* 7-8 intron-all exon ratio**	0.183580514
